# Cancer of the anal canal, a reality in the Colombian coffee region. Clinical-epidemiological review 2000–2019

**DOI:** 10.3332/ecancer.2021.1181

**Published:** 2021-02-09

**Authors:** Carlos Raúl Villegas Mejía, Manuel Villegas Jaramillo, Pedro Villegas Jaramillo

**Affiliations:** 1 Clinical Oncology and Radiotherapy, Oncology Service, Oncologists of West SAS, Caldas 170004641, Colombia; 2 University of Manizales, Health Faculty, School of Medicine, Manizales, Caldas 170004641, Colombia; a https://orcid.org/0000-0002-0103-6844; b https://orcid.org/0000-0001-8672-3370; c https://orcid.org/0000-0001-5445-4989

**Keywords:** anal cancer, anal canal, profile, epidemiology, review

## Abstract

**Introduction:**

Anal cancer is a rare pathology which has increased over the last few decades, and, therefore, gained importance for the quality of life of affected individuals. Thus, a review has been conducted in the Colombian coffee region (Departments of Caldas, Quindío y Risaralda) describing its behaviour and clinical-epidemiological profile.

**Materials and methods:**

Descriptive review of 437 patients of Western SAS Oncologists between January 2000 and December 2019 with a diagnosis of anal cancer.

**Results:**

62% of cases presented in women with a median age of 62 years, 30% in the sixth decade; centred at 65% in three main cities designated as capitals (Manizales, Pereira and Armenia); 62% as localised disease, with 40% stage II-A and 6% as initial metastasis; 29% presented positive ganglia, particularly N1a; squamous cell or epidermoid histology in 90%; 16% poorly differentiated; 5% related to Human Immunodeficiency Virus infection; localisation in the medial area of the anal canal in 63% of cases; 83% completed treatment, and 92% of them received chemotherapy/radiation therapy with 87% based on the Nigro protocol; finally, 11% presented with relapse in the liver in 10% of cases and 55% local.

**Conclusion:**

Four hundred and thirty-seven patients evaluated over 20 years with follow up at median 34.13 months (standard deviation 41.75) with median survival at later ages decreasing to 62% in patients older than 80 years, and differences in survival in localised disease at 78% in comparison to 46% in advanced metastasis. Finally, the overall 5-year survival rate is 69% with a median survival of 191 months in the study.

## Introduction

Anal cancer is a rare pathology and is considered infrequent, accounting for 1%–2.6% of digestive cancers and 2%–4% of anal-colorectal tumours [1], with increased incidence worldwide [2] and an increasing rate of 2.9%/year since 1992, according to The Surveillance, Epidemiology, and End Results Program of National Cancer Institute (SEER)-USA [3]. In Europe, nearly 2,000 men and 2,300 women are diagnosed each year [4].

According to the Global Cancer Observatory (GLOBOCAN), an agency belonging to the International Agency for Research on Cancer of the World Health Organisation, the global incidence rate is >1.2 per 100,000 women and 0.6–0.89 per 100,000 men. In the USA, the rate is 1 per 100,000 men and 1.8 women, with an overall rate of 1.4 per 100,000 inhabitants and mortality of 0.2 per 100,000 inhabitants. In comparison, in South America, the incidence is 0.57 per 100,000 for men and 1.20 per 100,000 in women with an overall rate of 0.92 per 100,000 inhabitants and a mortality rate of 0.19 per 100,000 inhabitants [5].

In reviewing South American cases, references are found from Brazil—a retrospective of 25 years with 49 cases [6]; from Venezuela—a report of results from 96 cases from 2000 to 2013 [7], and a previous report of 56 patients, and finally, in Chile, the mortality rate was reported at a minimum of 0.11 in 2003, increasing to 0.21 per 100,000 in 2010 [8].

In Colombia, according to GLOBOCAN, an incidence of 557 new cases was reported (0.55%), occupying 26th place, and a mortality rate of 84 cases (0.18%) with a prevalence of 1,395 cases (prop.2.82) in 2018: for Colombian statistics, a 15-year review effected at the Bogota National Cancer Institute shows 18% of cases [10], and a report of the results of the analysis of 82 patients of Oliveros R *et al* [11] published in 1990, treated by the gastroenterology service at the Bogota National Cancer Institute from 1935 to 1981.

With respect to local statistics, according to the Manizales Quinquennial Population Cancer Registry 2003–2007, the incidence is 0.8% (12 cases) in men and 0.6% (11 cases) in women, with 0.7% overall. During the 5-year period from 2008 to 2012, it was 0.5%, with an incidence rate of 0.9 per 100,000 inhabitants [12] and more specifically, in the area of the Caldas Department, a frequency of 0.7% is found (26th place) [13].

Anal cancer is an uncommon neoplasm; however, its incidence has recently increased due to Human Immunodeficiency Virus (HIV), making it the fourth most common cancer in this population with a general annual rate of 1 per 100,000 inhabitants to 35 per 100,000 inhabitants in HIV(+) men. Other factors include anal sex, promiscuity, immunosuppression, effects of Human Papillomavirus (HPV) attributed to HPV-16 serotype, the cause in both genders of 73% of cases, with the HPV-18 serotype attributed to 3.4%–7% [14]. According to the Centers for Disease Control and Prevention, these main serotypes [15] are present in 79% of anal cancers, attributed to the use and abuse of cigarettes, which causes an increase in cases in both sexes with worldwide predominance in males in this population [16]. The Colombian Coffee Sector (Caldas, Quindío and Risaralda Departments) does not escape this reality due to the scarcity of epidemiological data. In view of this, an analysis of the effects of anal cancer in the Western SAS Oncologists’ area of influence was performed to provide evidence of the magnitude of the problem, its clinical profile, epidemiology, treatment and finally, the results attained for this pathology.

## Materials and methods

A retrospective analysis was performed from the database of the Western SAS Oncologists, a private organisation dedicated to cancer awareness, treatment and monitoring in this geographical area, from 01/01/2000 to 31/12/2019, using International Disease Classification codes 10th edition (CIE-10) with C21.0, C21.1, C21.2 and C21.8 related to anal cancer [17]. The CIE-10 was employed as a base classification as the institutional standard for the classification of cancer patients who were examined, and with compromised histology as an exclusion criterion, thus raising doubts about anatomical location in the final selection of a population base for the analysis of 437 cases.

To begin, general clinical and demographic aspects were analysed (age, occupation, procedure, department, municipality, clinical stage, oncological classification by tumour, nodules and metastasis (approved by the 2017 investigatory team at tumour-node-metastasis (TNM) to avoid confusion and time variation), HIV, Karnofsky and symptom duration); anatomical variables (histology, histological grade, prognostic factors, anatomical location within the canal); therapeutic variables (surgery, type of non-surgical treatment, gap in radiation therapy, initial and rescue chemotherapy plans, radiation therapy dose and sessions, site of recurrence, time at recurrence, number of rescue chemotherapy treatments and a description of the therapy as completed or not) and finally, based on the results of intervention and survival (overall monitoring and survival according to different variables analysed). These factors may be analysed and defined as risk factors according to the results obtained. The date of diagnosis was analysed in 5-year increments to evaluate the patterns, age by 10- and 20-year period and the clinical stage as defined by the most recent classification of the American Joint Committee on Cancer (AJCC) from 2017 [18].

The histological grade was classified as well differentiated, moderately differentiated and poorly differentiated, defined by an institutional or external pathology team, and was based on the pathological findings following established guidelines for the speciality, which is outside the scope of this analysis. The treatment was based on surgery, radiation treatment, chemotherapy, radiation and chemotherapy together or none. Recurrence, or clinical evidence of tumour activity, was evaluated by the oncologist or, when in doubt clinically, based on a positive biopsy report confirming tumour activity following treatment, or without the necessity of a pathological study if a lesion, defined by paraclinical methods such as endoscopy or radiology, was present, with all of the foregoing occurring following a disease-free period of 12 months or more. The persistence of the tumour was defined as aforementioned, without a disease-free period, after completing the principle treatment. The gap variable was described as the time, in days, of radiation therapy interruption, with a goal of permitting functional recovery of the patient from generally morbidity from chemotherapy/radiation therapy [6]. Symptom duration is given in months.

Monitoring was defined as the period from the date of diagnosis to the final completed treatment, and survival was defined as the period from the end of treatment to remission or death. The final stage was determined using categories of alive or dead, sub-classified as active, lost and deceased, with or without tumour activity.

Qualitative variables were analysed as proportions, and quantitative variables are calculated using standard deviations. Survival calculations were performed using the Kaplan–Meier estimator and the log-Rank test. Statistical analysis was based on Epi Info 6.04™, Version 3.5.4, and IBM SPSS, Version 22^®^.

The project was approved by the Medical Director and the Research Department of Western SAS Oncologists, with the approval and supervision of the appropriate ethics committee, who permitted the inclusion of their business name in the publication. Given that there is no alternative therapeutic intervention or external information particular to the accepted management of these patients, it is considered a study without risk following Article 11 of the 1993 Resolution 8430 of the Colombian Ministry of Health, and current regulations pertaining to health research adhering to the Guide of the International Conference of Tripartite Harmonisation, Harmonisation of Good Clinical Practices and the Helsinki Declaration (Version 64a of the General Assembly, Fortaleza, Brazil, October 2013).

## Results

**Figure 1.**
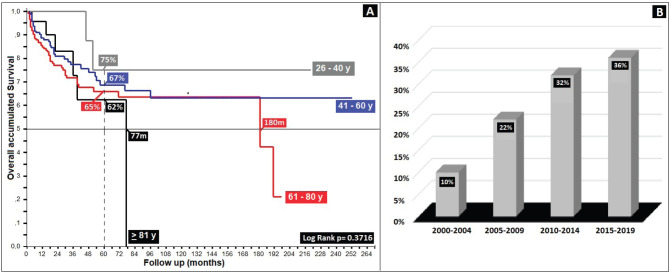
Age distribution in patients with anal cancer by age. (a): Distribution of survival by age groups in twenties. (b): Behaviour of anal cancer according to five-yearly.

Anal cancer constitutes 2% of colorectal anal tumours, presenting twice as often in females (62%) and tripling in frequency beginning in the fifth decade (Table 1), with survival diminishing as age increases, from 75% in the 26–40-year-old age group to 62% for ≥80 years, displaying an inverse relationship between age and survival (*p* = 0.37) (Figure 1).

**Figure 2.**
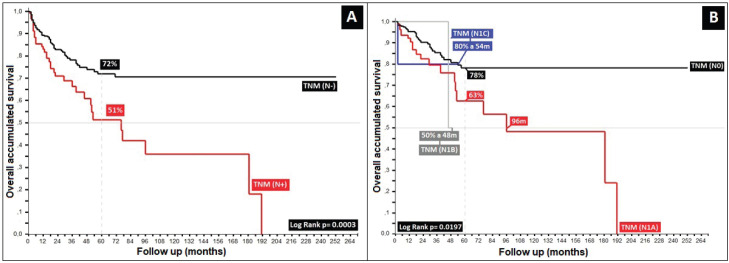
Distribution according to TNM (N) survival in anal cancer patients. (a): Distribution according to the presence of positive or negative nodes. (b): Anal cancer behaviour according to TNM (N).

**Figure 3.**
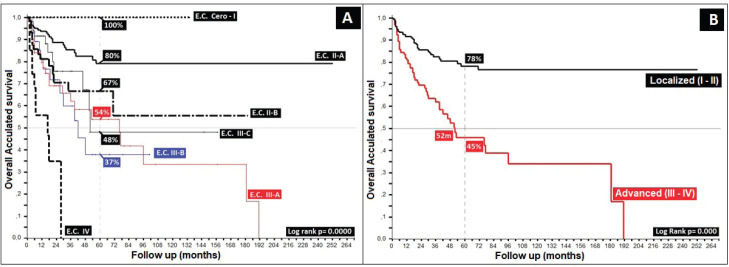
Distribution according to TNM (T) in patients with anal cancer. (a): Anal cancer behaviour according to TNM (T). (b): Distribution according to localised or advanced disease.

Regarding clinical staging, localised stages (E.C. II and TNM:T2) constituted 54%, with the sub-type II-A in the majority (40%); 26% with TNM N1a; and only 6% as initial metastasis, with 50% at the locoregional level and 13% of these at the bone level. Epidermoid histology was 90%. Differences in 5-year overall survival (_5_OS) between well- and poorly-differentiated were 10% (68% versus 58%, *p* = 0.05). 28% of the population presented with an adverse prognostic factor, i.e. the presence of ganglia demonstrated by our results and favouring negative ganglia versus positive (_5_OS: 72% versus 51%, *p* = 0.00), similar to comparisons of TNM N0 versus N1a, at 20% (*p* = 0.00), with differences of 12% between sub-types II-A and II-B (_5_OS: 78% versus 66%, *p* = 0.00), and defining sub-type II-B and TNM T4 (Figures 2 and 3) as the same as ulceration as the principle adverse prognostic factors with differences in comparison to those not presenting factors (*p* = 0.03). Medial location in the canal was 2/3 higher than lateral (_5_OS: 70% versus 55%, *p* = 0.56). Karnofsky ≥ 80% doubled in the 50%–70% survival group (70% versus 35%, *p* = 0.00) (Table 2).

**Figure 4.**
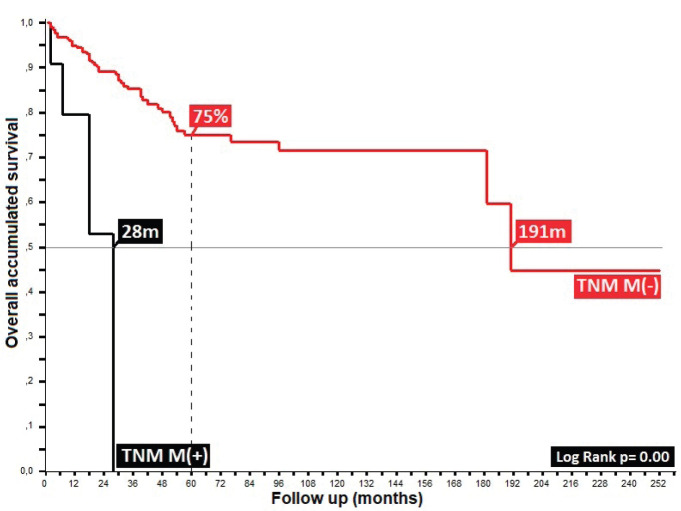
Distribution according to TNM (M) state in patients with anal cancer.

87% of the group who completed treatment received the Nigro protocol, in comparison to 6% receiving the Mayo protocol, 4% receiving Capecitabine/Mitomicina C (MMC) as a variant of the Nigro protocol and 3% receiving 5-Fluoropirimidina (5-FU)/Cisplatin (CDDP), with no statistically significant differences between Nigro, 5-FU/CDDP and Mayo (_5_OS: 75% versus 68% versus 62%, *p* = 0.34), though favouring the alternate protocol of Capecitabine/MMC in comparison to Nigro (91% versus 75%, *p* = 0.58). Comparing the protocols designated as second line, with overall rates of response nearing 70% and mOS of 12 months for 5-FU/Cisplatin, slightly less than Nigro (Figure 4). As with other adverse factors, status as described by the Karnofsky scale becomes, in the majority of neoplasms, a further prognostic factor.

The total dose for radiation therapy in the group who completed treatment was 49.08 Gys, with differences within the group of greater or less than 45.00 Gys, favouring the larger applied dose (_5_OS: 77% versus 68%, *p* = 0.01) (Table 3).

32% presented with recurrence, with a statistically significant difference in survival between persistence (17%) and recurrence (47%) (*p* = 0.00), with this difference being larger upon comparison between patients who did or did not present with recurrence (36% versus 90%, *p* = 0.00), along with differences between patients who completed treatment in comparison to those who did not: 52% in _5_OS and a mean survival (mOS) of 177 months, favouring the group who completed treatment, to ultimately generate an overall survival of 95%, 89%, 85%, 81% and 69% from the first to the fifth year, and an mOS of 191 months (Table 4).

## Discussion

Anal cancer is a rare pathology that is on the rise, reflected by the trend found starting at 10% in the first 5-year period (2000–2004) and reaching 36% in the final 5-year period (2015–2019), similar to that found by other authors in Europe [5, 19], the United States [1, 2], as well as South America [6–11]. Survival and frequency increased over the analysis period, beginning at 54% survival in the 5-year period from 2010 to 2014 and reaching 74% for the 5-year period from 2015 to 2019 (*p* = 0.07), and maintaining a ratio of male:female favouring women during the different 5-year periods, the ratio being 1.65 generally with survival favouring same (_5_OS: 68% versus 63%, *p* = 0.31) and (mOS: 190 versus 182 months, *p* = 0.32), similar to others [6, 7, 19–22], and a greater incidence of presentation in the population in their sixth decade [2, 6, 19].

The localised stage, described as presenting with lesions less than 5 cm, was found in 6%, with sub-type II-A being the most frequent (40%), similar to others [2, 18, 20, 22–24]. Metastatic disease originally presented in 6%, which accords with other authors who report frequencies of less than 10% [1, 2, 18], with statistically significant results regarding the tumour (TNM T, *p* = 0.01), ganglia compromise (TMN N, *p* = 0.00), as well as metastasis (TNM M, *p* = 0.00), corroborated by other authors, wherein the clinical stage as defined by tumour size, extension, ganglion compromise, and particularly the ganglion level, are adverse prognostic factors [20, 21, 25] (Table 5).

Similar to the literature world-wide, the epidermoid or squamous cell variety represented 90% [2, 15, 23], with 9% adenocarcinoma histology [7, 26–28], with the difference favouring the epidermoid type (_5_OS: 71% versus 24%, and mOS: 190 versus 28 months, *p* = 0.00), converting, as has been mentioned by others, to adenocarcinoma in an adverse prognostic histology [23, 26, 28, 29].

With respect to prognostic factors found in 28%, it can be concluded that positive ganglia, ulceration, adenocarcinoma-type histology, tumours ≥ 5 cm, TNM T4, obstruction, metastatic disease, and male gender, are considered, similarly to other authors, as factors that negatively affect survival [19–22, 30, 31], as was demonstrated by comparing negative or positive presence of ganglia (_5_OS: 72% versus 51%, *p* = 0.00) and the TNM N0 versus N1a sub-types in 20% (*p* = 0.00) [19–23, 25].

Initial presentation with TNM T as an adverse factor, particularly in tumours larger than 5 cm (T3), and T4 sub-classification (any size with vaginal, urethral or bladder compromise), corroborated by others, proved statistically significant (*p* = 0.00), generating results of _5_OS of 95%, 71%, 53% and 43% for T1, T2, T3 and T4, respectively, with differences between stages with smaller than 5 cm (T1 and T2) and larger (T3 and T4) of 10% in _5_OS of 100 months in mOS (*p* = 0.00), and regarding T3–T4, differences of 10% in _5_OS and 29 months in mOS among them, comporting with adverse factors in a non-statistically significant manner (*p* = 0.8215), possibly because T4 also included therein tumours ≥ 5 cm, as defined in T3 [19, 20, 22].

Regarding the most common clinical stage, II, presenting in 54%, differences are found in _5_OS of 12% between the II-A and II-B sub-types (*p* = 0.02), as also found by others [1–3], showing that the differences were essentially in tumour size (smaller or larger than 5 cm), defined in the 2017 AJCC and establishing the TNM II-B sub-type for future analysis as a risk factor for localised stages [20, 22, 24]. Data for _5_OS for stage Zero-I was 100%, with 54%, 38% and 48% for stages III-A, III-B and III-C, with mOS of 78, 42, 50 and 18 months for stages III-A, III-B, III-C and IV, respectively, and, finally, not reaching mOS in localised stages (Zero, I, II-A and II-B) (*p* = 0.00), showing once again the TNM T4 sub-type to be an adverse prognostic factor in comparison to ganglion compromise in tumours of smaller size II-A (T1–T2 with positive ganglia) [20, 32].

Ulceration presented as an adverse factor in _5_OS in 56%, similar to that reported by others, and constituting an adverse factor for consideration in future investigations, alongside node status (*p* = 0.00) and male gender (0.01) [33].

In the 6% of cases representing initial metastatic disease, the liver was the most affected site at 50%, similar to others, with data showing between 5% and 10% for metastatic disease [7, 19, 23, 29, 34] constituting an independent adverse prognostic factor with maximum survival of 28% in comparison to 75% for the group without metastasis, and mOS of 28 versus 191 months (*p* = 0.00), respectively, with differences resulting from the comparison of local disease with locally advanced/metastatic disease, such as the majority of the series wherein a regimen other than the Nigro protocol was proposed [35–38]**.**

Upon histological analysis, differences are found both in frequency (90% versus 9%) and in results favouring the epidermoid (_5_OS 71% versus 24% and mOS: 162 months, *p* = 0.00) [15, 16, 23, 28, 29].

Regarding HIV and its role in the development of anal cancer, 1% of women and 28% of men with anal cancer have an underlying infection, with this neoplasm occurring more in this population in comparison with the general population. In 4.6%, HIV was found to be present; a low number, but maintaining the relationship to male gender, similar to others [28, 29, 39, 40], with marked numerical differences between HIV (+) and HIV (−) populations, favouring the negative (_5_OS: 9% versus 66%, *p* = 0.08), without the ability to compare the larger series in which they were excluded [30, 32, 33, 41], and with contradictory results in different studies depending upon whether they were included in the analysed population [14, 42].

**Figure 5.**
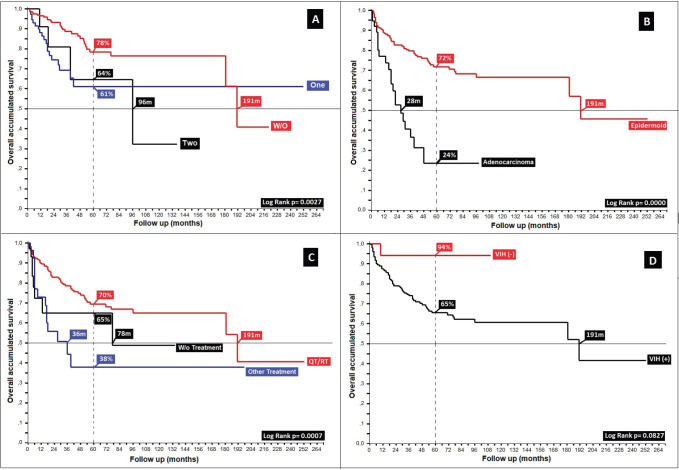
Distribution of patients with anal cancer according to different variables. (a): Distribution according to the presence of prognostic adverse factors. (b): Anal cancer behaviour according to histology. (c): Distribution according to the non-surgical treatment used. (d): Anal cancer behaviour based on HIV.

Similar to other authors, functional status is shown as a prognostic factor with differences in mOS between patients with Karnofsky ≤ 40%, 50%–70% and ≥80% of 8 and 180 months (10, 18 and 190 months, *p* = 0.00), respectively (Figure 5) [19, 41].

89% completed treatment based on concomitant chemotherapy/radiation therapy, of whom 87% received the Nigro protocol [35], generating differences upon grouping them as chemotherapy/radiation therapy, other treatments (surgery, radiation therapy, chemotherapy) and no treatment (*p* = 0.00). In the group who completed treatment, the differences between patients treated with Nigro (87%) compared to 5-FU/Cisplatin and others were _5_OS: 72%, 62% and 56% (*p* = 0.10). This further confirms that expressed by multiple authors since the Nigro protocol was first presented at the Meeting of the American Proctologic Society in Detroit in June of 1973, and published in 1974 [35]. The improvement with the Nigro protocol, which has become the standard treatment, shows minimal differences in comparison to 5FU/CDDP according to reports by others [29, 31, 37, 38], and corroborated by our results from the Nigro protocol versus 5-FU/CDDP (_5_OS 71% versus 48%, *p* = 0.04) and a difference of 151 months in mOS favouring the Nigro protocol (mOS 191 versus 40 months) [31, 32, 37, 38].

Analysing the other protocols employed, 5% received the Mayo protocol, with the difference (_5_OS: 55% versus 71%, *p* = 0.06) in favour of Nigro, and 4% received an alternative protocol, Capecitabine/MMC, with results showing slight benefit to the alternative protocol (_5_OS: 91% versus 75%, *p* = 0.58), with no statistically significant difference in concordance with different authors based on non-inferior results, such as alternative therapies in patients who were not candidates for Nigro [23, 29, 43, 44] for medical reasons, co-morbidities or lack of availability of infusion pumps to administer the Nigro protocol.

**Figure 6.**
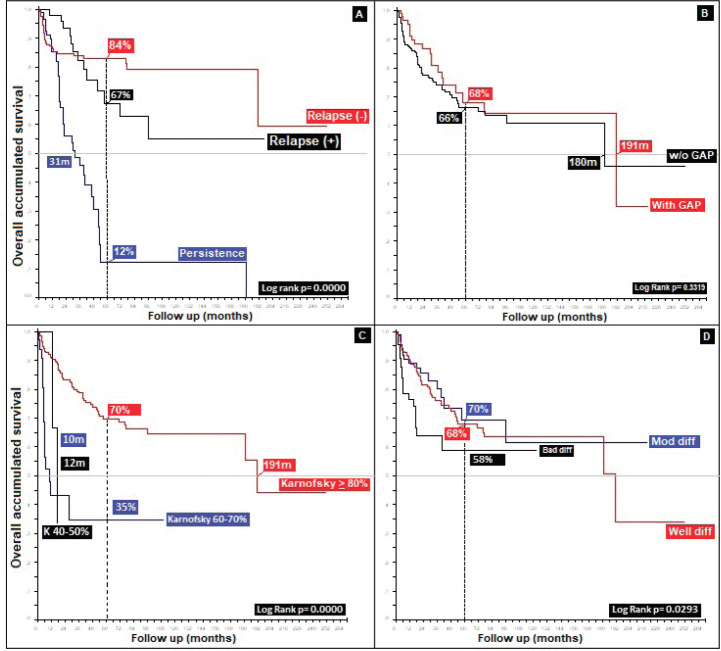
Distribution of patients with anal cancer according to different variables. (a): Distribution according to the presence of relapse. (b): Anal cancer behaviour according to the radiation GAP. (c): Distribution according to Karnofsky status. (d): Anal cancer behaviour according to histological grade.

Radiation therapy, specifically for the group who completed treatment (76%), gave differences in favour of a higher dose, 45.00 Gys (_5_OS: 68% versus 77%, *p* = 0.01). Analysis of different applied doses shows, similar to others, that better survival is not reflective of increased dose [45], as is demonstrated by </> 60.00 Gys, with the difference favouring the group receiving the lower dose (_5_OS: 38% and mOS: 140 months, *p* = 0.57), defining the optimal dose for localised stages as between 45.00 and 50.40 Gys, and possibly for more advanced stages according to TNM T and N(+), a larger dose is required, up to 54.00–60.00 Gys [19]. With respect to a gap during radiation therapy, this presented in 24%, leading to differences of 2% in _5_OS and 11 months in mOS, favouring the use of a gap in a non-statistically significant manner (*p* = 0.33), contrary to others who conclude that poorer outcomes are reflective of a gap [34, 46] (Figure 6).

**Figure 7.**
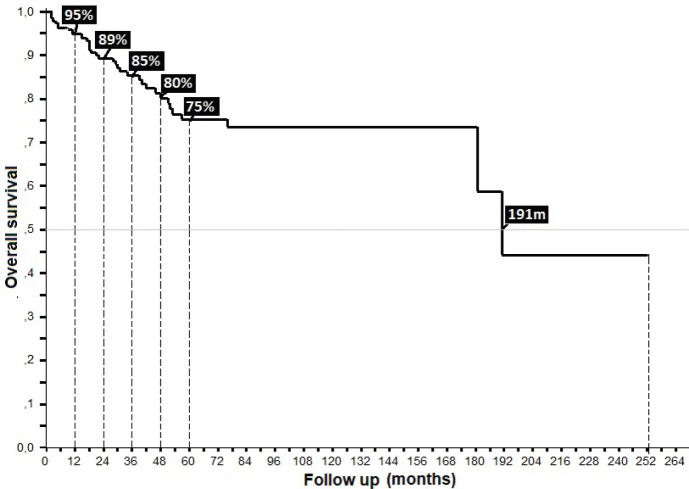
Overall survival of anal cancer.

Recurrence was noted in 11% at an average of 39.83 months (standard deviation 35, 39); 55% at a local level and 10% in the liver, similar to what others have noted [47, 48]. Persistence in 21% generated differences in _5_OS between the presence of persistence, recurrence and no recurrence of 12%, 67% and 84%, respectively, (*p* = 0.00), and, upon grouping the data as recurrence or no recurrence, the differences remain (40% versus 84%, *p* = 0.00). Regarding rescue therapy, similar to others, the practice of only making a therapeutic attempt leads to differences of 30% in _5_OS and 140 months in mOS (*p* = 0.13), in comparison with not attempting it. Finally, with respect to rescue chemotherapy, the protocols are based on 5-FU/CDDP (F-FU/Cisplatin), followed by Nigro (5-FU/Mitomycin) the overall population, favouring 5-FU/CDDP as the rescue. In modern series with few patients, triconjugate protocols are employed; others are based on taxanes, platins, and, finally, based on targeted and immunomodulatory therapy, without reaching an ideal rescue protocol to replace 5-FU/CDDP [36, 37, 43, 47–49], as shown in (Figure 7), with 82% of the patients categorised as completing treatment and classifying them on the basis of whether or not recurrence occurred, 32% presented with this event, with 53% receiving rescue treatment and completing it in 96% of cases.

## Conclusion

Anal cancer is increasing in the Colombian Coffee Area, with localised disease comprising the majority of cases, and the highest probabilities of cure ultimately defined by an epidemiological profile of women between 50 and 70 years of age, homemakers, from urban areas, initial stage of II-A, being T2, without ganglia and without initial metastasis. In the case of presenting ganglia of type N1a, with well-differentiated epidermoid histology and ulceration, 5% with HIV, the localised lesion found in the medial portion of the anal canal, a good functional state represented by Karnofsky ≥ 80% and symptoms over 6–12 months, who could receive treatment with chemotherapy/radiation therapy on the Nigro protocol with no gap, with a radiation therapy dose greater than 45.00 Gys. Despite the foregoing, recurrence at the locoregional and/or hepatic level occurred in 32% after 3 years of monitoring, and was principally treated with surgery, radiation therapy or chemotherapy/radiation therapy using the 5-FU/CDDP protocol reaching an average monitoring period of 39.42 months to ultimately generate an overall survival of 95%, 89%, 85%, 81% and 69% from the first through fifth years of follow-up. For the aforementioned, it was considered that the work complied with the objectives formed from the start to show the behaviour of this neoplasm in the coffee region.

## Conflicts of interest

The authors declare no conflicts of interest of any kind in the collection, analysis of information or the results from an academic, labour or economic point of view.

## Funding statement

The concepts presented herein are the sole responsibility of the authors, and the work was entirely financed by the investigators’ own resources without the intervention of any company or entity.

## Figures and Tables

**Table 1.** Clinical and demographic characteristics.

Variable	Category		Male (%)		Female (%)		Global	*p*
			Frec	(%)	Frec	(%)	*n* (%)	
**Gender**			165	38	272	62	437 (100)	0.31
Dx (year**)**	2000–2004		18	4	27	6	45 (10)	0.07
	2005–2009		31	7	65	15	96 (22)	
	2010–2014		55	13	85	19	140 (32)	
	2015–2019		61	14	95	22	156 (36)	
Age	20–40		10	2	8	2	18 (4)	0.37
	41–60		67	15	103	24	170 (39)	
	61–80		75	17	147	34	222 (51)	
	81 or more		13	3	14	3	27 (6)	
			Median: 61.7/DE: 12.7		Median: 62.7/DE: 11.7		Median: 62.3/DE: 12.1	
	20–30		2	0	1	0	3 (1)	0.59
	31–40		8	2	7	2	15 (3)	
	41–50		16	4	34	8	50 (11)	
	51–60		51	12	69	16	120 (27)	
	61–70		51	12	82	19	133 (30)	
	71–80		24	5	65	15	89 (20)	
	81–90		9	2	13	3	22 (5)	
	90 or more		4	1	1	0	5 (2)	
Occupation	Various		64	15	247	57	311 (71)	0.43
	Auxiliary		28	6	6	1	34 (8)	
	Unemployed		31	7	1	0	32 (7)	
	Pensioner		22	5	7	2	29 (7)	
	Independent		11	3	3	1	14 (3)	
	Professional		8	2	2	0	10 (2%	
	Téchnical		1	0	6	1	7 (2)	
Zone	Urban		155	35	265	61	420 (96)	0.76
	Rural		10	2	7	2	17 (4)	
Department	Caldas		76	17	101	23	177 (41)	0.00
	Risaralda		43	10	82	19	125 (29)	
	Quindío		36	8	58	13	94 (21)	
	Valle		10	2	31	7	41 (9)	
Department/City	Caldas	Manizales	51	12	51	12	113 (64)	0.00
		Chinchina	4	1	4	1	13 (7)	
	Risaralda	Pereira	27	6	27	6	76 (61)	0.48
		Dosquebradas	12	3	12	3	31 (25)	
	Quindío	Armenia	43	10	22	5	65 (69)	0.57
		Calarca	3	1	6	1	9 (10)	
	Valle	Cartago	14	13	2	0	16 (39)	0.35
		La Victoria	3	1	0	0	3 (7)	

Dx; diagnostic, Frec; frequency

**Table 2.** Clinical and demographic characteristics.

Variable	Category		Male (%)		Female (%)		Global	*p*
			Frec	(%)	Frec	(%)	*n* (%)	
Clinical Stage	Cero		4	1	4	1	8 (2)	0.00
	I		8	2	18	4	26 (6)	
	II		84	19	153	35	238 (54)	
		II-A	59	14	114	26	173 (40)	
		II-B	24	5	41	9	65 (14)	
	III		58	13	83	20	141 (33)	
		III-A	29	7	26	6	55 (13)	
		III-B	6	1	22	6	28 (7)	
		III-C	23	5	35	8	58 (13)	
	IV		12	3	12	3	24 (6)	
TNM T	In situ		5	1	5	1	10 (2)	0.00
	T1		10	2	19	2	29 (7)	
	T2		89	20	146	33	235 (54)	
	T3		45	10	68	16	113 (26)	
	T4		16	4	34	8	50 (11)	
TNM N	N(−)		105	24	206	47	311 (71)	0.00
	N(+)		60	14	66	15	126 (29)	
TNM subtype N	No		105	24	206	47	311 (72)	0.00
	N1A		52	12	63	14	115 (26)	
	N1B		3	1	2	0	5 (1)	
	N1C		5	1	1	0	6 (1)	
TNM M	Mo		153	35	260	59	413 (94)	0.00
	M1		12	3	12	3	24 (6)	
Histology	Epidermoid		139	32	256	59	395 (90)	0.00
	Adenocarcinoma		24	5	14	3	38 (9)	
	Melanoma		0	0	3	1	3 (1)	
	Kaposi		1	0	0	0	1 (0)	
Grade	Well diff.		100	23	177	41	277 (63)	0.05
	Mod. Diff.		34	8	56	13	90 (21)	
	Bad diff.		31	7	39	9	70 (16)	
Fx Px	W/o		110	25	206	47	316 (72)	0.03
	Ulceration		43	10	38	9	81 (18)	
	Obstruction		13	3	22	5	35 (8)	
	Linfovascular invasion		1	0	3	1	4 (1)	
Nro Fx Px	Zero		112	26	204	47	316 (72)	0.00
	One		41	9	57	13	98 (22)	
	Two		12	3	11	3	23 (6)	
HIV	Yes		18	4	2	1	20 (5)	0.08
	No		147	34	270	62	417 (95)	
Site in canal	Medial		120	27	157	36	277 (63)	0.56
	Lateral		27	6	75	17	102 (23)	
	Rectal ext		12	3	26	6	38 (9)	
	Anal margin		6	1	14	3	20 (5)	
Karnofsky	80%–90%		143	33	255	58	398 (91)	0.00
	60%–70%		19	4	17	4	36 (8)	
	≤50%		3	1	0	0	3 (1)	
Symptoms	<3 months		18	4	15	3	33 (8)	0.02
	3–6 months		68	16	105	24	173 (40)	
	6–12 months		70	16	146	33	216 (49)	
	>12 months		9	2	6	1	15 (3)	
			Median: 8.5/DE: 6.0/R: 1–48		Median: 7.4/DE: 3.4/R: 2–36		Median: 7.8/DE: 4.5/R: 1–48	

Dx; diagnostic, Frec; frequency, T; tumour, N; node, M; metastasis, Diff; differentiated, Fx; factors, Px; prognostic

**Table 3.** Intervention characteristics.

Variable	Category		Male (%)		Female (%)		Global	*p*
			Frec	(%)	Frec	(%)	*n* (%)	
Surgery	No		148	34	236	54	384 (88)	0.29
	Pal colostomy		7	2	16	3	23 (5)	
	RAP		6	1	10	3	16 (4)	
	Local resection		4	1	9	2	13 (3)	
	RAUB		0	0	1	0	1 (0)	
Non surgical treatment	QT/RT		129	29	239	55	368 (84)	0.00
	None		22	5	21	5	43 (10)	
	RT		9	2	8	2	17 (4)	
	QT		5	1	4	1	9 (2)	
GAP (days) *n* = 368 (QT/RT)	Yes		24	7	63	17	87 (24)	0.33
	Yes		105	28	176	48	281 (76)	
			Median: 2.9/DE: 8.2/R: 0–45		Median: 4.3/DE: 10.9/R: 0–106		Median: 4.3/DE: 10.9/R: 0–106	
Initial Qt QT/RT *n* = 368	NIGRO		116	31	197	54	313 (85)	0.02
	MAYO		5	1	14	4	19 (5)	
	Xeloda/MMC		2	0	13	4	15 (4)	
	5-FU/CDDP		3	1	9	2	12 (3)	
	Other/Mixed:		1	0	9	2	10 (2)	
		Capecitabine	2	0	3	1	5 (1)	
		CDDP	1	0	2	1	3 (1)	
Radiotherapy *n* = 331 QT/RT Full Tto	<45.00 Gys		41	12	81	24	122 (37)	0.01
	>45.00 Gys		75	23	134	40	209 (63)	
							
	<45.00 Gys		41	12	81	24	122 (37)	0.19
	45.00–50.40 Gys		43	13	92	28	135 (41)	
	50.40–55.00 Gys		15	5	13	4	28 (9)	
	55.00–60.00 Gys		10	3	22	7	32 (10)	
	>60.00 Gys		7	2	7	2	14 (4)	
DT RT (Gys)	General		Median: 47.23		Median: 45.70		Median: 46.24	NA
	Complete Tto		Median: 49.86		Median: 49.03		Median: 49.08	
No. sessions	Complete Tto		Median: 26.42/DE: 3.24/R: 20–37		Median: 25.39/DE: 3.39/R: 5–35		Median: 25.75/DE: 3.37/R: 5–37	

Frec; frequency, Pal; palliative, RAP; abdominoperineal resection, RAUB; anterior ultra low resection, QT/RT; concomitant chemo-radiotherapy, QT; chemotherapy, RT; radiotherapy, GAP; rest time on RT treatment, Gys; absorbed radiation dose unit, DT; total dose, DE; standard deviation, R; range, SNC; central nervous system, T; time; Cx; surgery, Tto; treatment

**Table 4.** Intervention characteristics.

Variable	Category	Male (%)		Female (%)		Global	*p*
		Frec	(%)	Frec	(%)	*n* (%)	
Relapse	No	112	26	183	42	296 (68)	0.00
	Persistence	34	8	57	13	93 (21)	
	Yes	19	4	32	7	48 (11)	
Relapse site *n* = 213 events	Local	48	23	69	34	117 (55)	0.00
	Liver	8	4	14	6	22 (10)	
	Osseous	3	1	11	5	14 (7)	
	Lung	5	2	10	5	13 (6)	
	Inguinal	3	1	7	4	10 (5)	
	Vagina	0	0	8	4	8 (4)	
	SNC	3	1	2	1	5 (2)	
	Supraclavicular	2	1	2	1	4 (2)	
	Retroperitoneum	1	0	3	2	4 (2)	
	Carcinomatosis	1	0	2	2	3 (2)	
	Other	3	1	6	3	9 (4)	
Relapse T	(meses)	Median: 34.23/DE: 29.96		Median: 44.18/DE: 39.10		Median: 39.83/DE: 35.39/R: 13–149	
Relapse treatment	None	134	31	228	52	362 (83)	0.54
	Mixed	10	2	15	3	25 (6)	
	RT	7	2	8	2	15 (3)	
	QT	6	1	8	2	14 (3)	
	Cx	5	1	9	2	14 (3)	
	QT/RT	3	1	4	1	7 (2)	
QT rescue *n* = 60 events	Other/mixed	6	10	10	17	16 (27)	0.27
	5-FU/CDDP	7	12	8	13	15 (25)	
	Capecitabine	2	3	6	10	8 (13)	
	XelOx	3	5	4	7	7 (12)	
	NIGRO	1	2	5	8	6 (10)	
	Mayo	2	3	4	7	6 (10)	
	5-FU	1	2	0	0	1 (2)	
No. schemes of QT	0	34	8	27	6	61 (14)	0.00
	1	117	27	218	50	335 (77)	
	2	10	2	21	5	31 (7)	
	3	3	1	4	1	7 (2)	
	4	1	0	2	1	3 (1)	
Complete Tto	Yes	116	32	215	58	331 (90)	0.00
QT/RT *n* = 368	No	13	4	24	6	37 (10)	

Frec; frequency, Pal; palliative, RAP; abdominoperineal resection, RAUB; anterior ultra low resection, QT/RT; concomitant chemo-radiotherapy, QT; chemotherapy, RT; radiotherapy, GAP; rest time on RT treatment, Gys; absorbed radiation dose unit, DT; total dose, DE; standard deviation, R; range, SNC; central nervous system, T; time, Cx; surgery, Tto; treatment

**Table 5.** Intervention and survival results.

Variable			Male		Female		Global
			Frec	%	Frec	%	
Follow up	Median: 31.27/DE: 37.61/R: 0–197			Median: 35.84/DE: 44.0/R: 0–252			Median: 34.13/DE: 41.77/R: 0–252
Outcome	Alive		126	29	216	49	342 (78)
	Dead		39	9	56	13	95 (22)
Overall survival	1st year	2nd year	3rd year	4th year	5th year	Median	
	95%	89%	85%	81%	69%	191 months	
QT scheme		NIGRO	Capecitabine/MMC	5-FU/CDDP	MAYO	*p* = 0.42	
	_5_OS	75%	91%	69%	62%		
	mOS	191 months	NA	96 months	NA		
Stage	IV stage	1st year	2nd year	Maxim	Median		
		55%	35%	35% at 28 months	17 months		
Age	Groups	26–40 years	41–60 years	61–80 years	>80 years	*p* = 0.37	
		74%	68%	66%	62%		
**Type**	**Variable**	_5_**OS**	**mOS (months)**	**Variable**	_5_**OS**	**mOS (months)**	*p*
Clinical stage	Cero-I	100%	NA				0.00
	II	75%	NA				
				II-A	78%	NA	
				II-B	66%	NA	
	III	50%	53				
				III-A	54%	73	
				III-B	38%	42	
				III-C	48%	50	
	IV	0%	17				
	**Variable**	**_5_OS**		**mOS (months)**		***p***	
	Localised	78%		50		0.00	
	Advanced	46%		NA			
TNM T	In situ	100%		NA		0.00	
	T1	94%		NA			
	T2	72%		191			
	T3	53%		70			
	T4	43%		50			
TNM N	N(−)	71%		NA		0.00	
	N(+)	51%		75			
TNM subtype N	N0	72%		NA		0.00	
	N1A	52%		75			
	N1B	40% at 49 months		49			
	N1C	62% at 50 months		(-)			
TNM M	M0	68%		NA		0.00	
	M1	34% at 28 months		14			
Non surgical	QT/RT	70%		191		0.00	
treatment	Other	65%		75			
	None	38%		38			
Histology	Epidermoid	77%		191		0.00	
	Adenocarcinoma	35%		40			

Frec; frequency, DE; standard deviation, R; range, SV; survival, _5_OS; 5-year overall survival, mOS; median survival, m; months, T; tumour, N; nodes, M; metastasis, Tto; treatment, Qx; surgical, QT/RT; concomitant chemo/radiotherapy

## References

[1] Siegel RL, Miller KD, Jemal A (2020). Cancer statistics. CA Cancer J Clin.

[2] National Comprehensive Cancer Network (NCCN) NCCN clinical practice guidelines in oncology. https://www.nccn.org/professionals/physician_gls/pdf/anal.pdf.

[3] Shiels MS, Kreimer AR, Coghill AE (2015). Anal Cancer Incidence in the United States, 1977–2011: distinct patterns by histology and behavior. Cancer Epidemiol Biomark Prev Publ Am Assoc Cancer Res Cosponsored Am Soc Prev Oncol.

[4] Rogers JE, Eng C (2017). Pharmacotherapy of anal cancer. Drugs.

[5] http://gco.iarc.fr/today/home.

[6] Parra R, Brito A, Rocha J, Féres O (2007). Retrospective analysis of patients with anal tumors diagnosed at the school of medicine of Ribeirão Preto Hospital and clinics (HC-FMRP), between 1979 and 2004 and literature review. Rev Bras Coloproctologia.

[7] Vera SL, Lafee NU, Colmenares OL (2015). Carcinoma de ano tratamiento combinado radioterapia y quimioterapia 13 años de experiencia. Rev Venezolana Oncol.

[8] https://www.researchgate.net/publication/307930008_Situacion_del_cancer_en_chile_2000_-_2010?enrichId=rgreq-0339a24473f661757eb6d219e2aeb97b-XXX&enrichSource=Y292ZXJQYWdlOzMwNzkzMDAwODtBUzo0MDQyNTk3MTY3ODAwMzJAMTQ3MzM5NDQzNTYwNg.

[9] https://gco.iarc.fr/today/data/factsheets/populations/170-colombia-fact-sheets.pdf.

[10] Colombia, Ministerio de Salud (2001). Guías de práctica clínica en enfermedades neoplásicas.

[11] Oliveros R, Rey-León C, Olarte H (1990). Carcinoma del ano experiencia con 82 pacientes. Rev Col Cir.

[12] Lopez GA (2012). Registro poblacional de cáncer de Manizales-Incidencia y mortalidad 2003–2007 Informe final de investigación.

[13] Villegas CR, Chacón JA, Cardona JP (2012). Perfil clínico epidemiológico de los pacientes con cáncer tratados en una Institución de tercer nivel. Manizales, Colombia, 1995–2004. Colomb Med.

[14] Wang C-Ch J, Sparano J, Palefsky JM (2017). Human immunodeficiency virus/aids, human papillomavirus, and anal cancer. Surg Oncol Clin N Am.

[15] Hoff PM, Coundry R, Venchiarutti CM (2017). Pathology of anal cancer. Surg Oncol Clin N Am.

[16] Symer MM, Yeo HL (2018). Recent advances in the management of anal cancer [version 1; peer review: 2 approved]. F1000Res.

[17] Ramos M-V AJ, Vázquez-Barquero JL, Herrera Castanedo S (2002). CIE-10 (I): Introducción, historia y estructura general. Pápeles Médicos.

[18] Amin MB, Greene FL, Edge SB (2017). The Eighth Edition AJCC Cancer Staging Manual: Continuing to build a bridge from a population‐based to a more “personalized” approach to cancer staging. CA Cancer J Clin.

[19] Glynne-Jones R, Nilsson PJ, Aschele C (2014). Anal cancer: ESMO-ESSO-ESTRO Clinical Practice Guidelines for diagnosis, treatment and follow-up. Ann Oncol.

[20] Gunderson LL, Moughan J, Ajani JA (2013). Anal carcinoma: impact of TN category of disease on survival, disease relapse, and colostomy failure in US Gastrointestinal Intergroup RTOG 98-11 phase 3 trial. Int J Radiat Oncol Biol Phys.

[21] Ajani JA, Winter KA, Gunderson LL (2010). Prognostic factors derived from a prospective database dictate clinical biology of anal cancer the intergroup trial (RTOG 98-11). Cancer.

[22] Gunderson LL, Jessup JM, Sargent DJ (2010). AJCC Hindgut Taskforce. Revised TN categorization for colon cancer based on national survival outcome data. J Clin Oncol.

[23] Cummings BJ, Ajani JA, Swallow CJ, DeVita VT, Lawrence TS, Rosemberg SA (2008). Cancer of the anal región. Cancer: Principles & Practice of Oncology.

[24] Goffredo P, Garancini M, Robinson TJ (2018). A National-Level Validation of the New American Joint Committee on Cancer 8th Edition Subclassification of Stage IIA and B Anal Squamous Cell Cancer. Ann Surg Oncol.

[25] Lewis GD, Haque W, Butler EB (2019). Survival outcomes and patterns of management for anal adenocarcinoma. Ann Surg Oncol.

[26] Anwar S, Welbourn H, Hill J (2013). Adenocarcinoma of the anal canal – a systematic review. Colorectal Dis.

[27] Marquez MF, Velasco AFJ, Belda LR (2013). Adenocarcinoma del canal anal. Revision de conjunto.

[28] https://mocbrasil.com/es/moc-tumores-solidos/cancer-gastrointestinal/12-ano/.

[29] David P, Ryan DP, Willett CG (2020). UpToDate: classification and epidemiology of anal cancer. Anal Cancer.

[30] Northover J, Glynne-Jones R, Sebag-Montefiore D (2010). Chemoradiation for the treatment of epidermoid anal cancer: 13-year follow-up of the first randomised UKCCCR Anal Cancer Trial (ACT I). Br J Cancer.

[31] James RD, Glynne-Jones R, Meadows HM (2013). Mitomycin or cisplatin chemoradiation with or without maintenance chemotherapy for treatment of squamous-cell carcinoma of the anus (ACT II): a randomised, phase 3, open-label, 2×2 factorial trial. Lancet Oncol.

[32] Gunderson LL, Winter KA, Ajani JA (2012). Long-term update of US GI Intergroup RTOG 98-11 phase III trial for anal carcinoma: survival, relapse, and colostomy failure with concurrent chemoradiation involving fluorouracil/mitomycin versus fluorouracil/cisplatin. J Clin Oncol.

[33] Bartelink H, Roelofsen F, Eschwege F (1997). Concomitant radiotherapy and chemotherapy is superior to radiotherapy alone in the treatment of locally advanced anal cancer: results of a phase III randomized trial of the European Organization for Research and Treatment of Cancer Radiotherapy and Gastrointestinal Cooperative Groups. J Clin Oncol.

[34] Ben-Josef E, Moughan J, Ajani JA (2010). Impact of overall treatment time on survival and local control in patients with anal cancer: a pooled data analysis of radiation therapy oncology group trials 87-04 and 98-11. J Clin Oncol.

[35] Nigro ND, Vaitkevicius VK, Considine B (1974). Combined therapy for cancer of the anal canal. Dis Colon Rectum.

[36] Kim Richard, Byer J, Fulp WJ (2014). Carboplatin and paclitaxel treatment is effective in advanced anal cancer. Oncology.

[37] Olivatto LO, Cabral V, Rosa A (2011). Mitomycin-C– or cisplatin-based chemoradiotherapy for anal canal carcinoma: long-term results. Int J Radiat Oncol Biol Phys.

[38] Gunderson LL, Winter K A, Ajani JA (2012). Long-term update of US GI Intergroup RTOG 98-11 phase III trial for anal carcinoma: survival, relapse, and colostomy failure with concurrent chemoradiation involving fluorouracil/mitomycin versus fluorouracil/cisplatin. J Clin Oncol.

[39] Cataño CJC (2004). Cáncer anal en la era del VIH: papel de la citología anal IATREIA.

[40] Shiels MS, Pfeiffer RM, Chaturvedi AK (2012). Impact of the HIV epidemic on the incidence rates of anal cancer in the United States. J Natl Cancer Inst.

[41] Tournier-Rangeard L, Mercier M, Peiffert D (2008). Radiochemotherapy of locally advanced anal canal carcinoma: Prospective assessment of early impact on the quality of life (randomized trial ACCORD 03). Radiother Oncol.

[42] Grew D, Bitterman D, Leichman CG (2015). HIV infection is associated with por poor outcomes for patients with anal cancer in the highly active antiretroviral therapy era. Dis Colon Rectum.

[43] Glynne-Jones R, Meadows H, Wan S (2008). EXTRA—a multicenter phase II study of chemoradiation using a 5 day per week oral regimen of capecitabine and intravenous mitomycin C in anal cancer. Int J Radiat Oncol Biol Phys.

[44] Peixoto RD’A, Wan DD, Schellenberg D (2016). A comparison between 5-fluorouracil/mitomycin and capecitabine/ mitomycin in combination with radiation for anal cancer. J Gastrointest Oncol.

[45] Prasad RN, Elson J, Kharofa J (2018). The effect of dose escalation for large squamous cell carcinomas of the anal canal. Clin Transl Oncol.

[46] Weber DC, Kurtz JM, Allal AS (2001). The impact of gap duration on local control in anal canal carcinoma treated by split-course radiotherapy and concomitant chemotherapy. Int J Radiat Oncol Biol Phys.

[47] Akbari RP, Paty PB, Guillem JG (2004). Oncologic outcomes of salvage surgery for epidermoid carcinoma of the anus initially managed with combined modality therapy. Dis Colon Rectum.

[48] Bentzen AG, Guren MG, Wanderås EH (2012). Chemoradiotherapy of anal carcinoma: survival and recurrence in an unselected national cohort. Int J Radiat Oncol Biol Phys.

[49] Garg MK, Zhao F, Sparano JA (2017). Cetuximab plus chemoradiotherapy in immunocompetent patients with anal carcinoma: a phase II Eastern Cooperative Oncology Group–American College of Radiology Imaging Network Cancer Research Group Trial (E3205). J Clin Oncol.

